# Antiviral and protective effect of small interfering RNAs against rift valley fever virus in vitro

**DOI:** 10.1007/s11033-023-08455-9

**Published:** 2023-05-25

**Authors:** Engy. M. Ahmed, Abeer. A. Boseila, Amro S. Hanora, Samar. M. Solyman

**Affiliations:** 1 Egyptian Drug Authority (EDA), Giza, Egypt; 2 Microbiology & Immunology Department, College of Pharmacy, Suez Canal University, Ismailia, Egypt; 3 Microbiology & Immunology Department, Faculty of Pharmacy, Sinai University Kantara branch, Ismailia, Egypt

**Keywords:** Rift valley fever virus (RVFV), Small interfering RNA (siRNA), Vero cells, RT-PCR, Endpoint assay, Western blot

## Abstract

**Background:**

Rift Valley Fever Virus (RVFV) is an arbovirus, a zoonotic disease that resurfaces as a potential hazard beyond geographic boundaries. Fever that can proceed to encephalitis, retinitis, hemorrhagic fever, and death is the main manifestation observed in human infections. RVFV has no authorized medication. The RNA interference (RNAi) gene silencing pathway is extremely well conserved. By targeting specific genes, small interfering RNA (siRNA) can be used to suppress viral replication. The aim of this study was to design specific siRNAs against RVFV and evaluate their prophylactic and antiviral effects on the Vero cells.

**Methods and results:**

Various siRNAs were designed using different bioinformatics tools. Three unique candidates were tested against an Egyptian sheep cell culture-adapted strain BSL-2 that suppressed RVFV N mRNA expression. SiRNAs were transfected a day before RVFV infection (pre-transfection), and 1 h after the viral infection (post-transfection), and were evaluated to detect the silencing activity and gene expression decrease using real-time PCR and a TCID50 endpoint test. The degree of N protein expression was determined by western blot 48 h after viral infection. D2 which targets the (488–506 nucleotides), the middle region of RVFV N mRNA was the most effective siRNA at 30 nM concentration, it almost eliminates N mRNA expression when utilized as antiviral or preventive therapy. siRNAs had a stronger antiviral silencing impact when they were post-transfected into Vero cells.

**Conclusion:**

Pre and post-transfection of siRNAs significantly reduced RVFV titer in cell lines, offering novel and potentially effective anti-RVFV epidemics and epizootics therapy.

## Introduction

The Rift Valley Fever Virus (RVFV), is a zoonotic arthropod pathogen that produces epidemics and epizootics in humans and domestic animals. RVFV has been categorized as a Category A priority pathogen besides being an overlap-select agent [1]. RVFV infection can result in abortion storms, with a 100% fatality rate in pregnant ewes, and a catastrophic economic impact on livestock [2]. Human infection results in fever, encephalitis, retinitis, hemorrhagic fever, and mortality [3, 4]. Direct contact with body fluids, infected animal tissues, intake of contaminated animal products [5], and mosquito bites all contribute to transmission. RVFV is endemic in Africa since Egypt has suffered from many outbreaks that seriously impacted humans and livestock [5]. Low levels of RVFV were reported in some areas in 2017, which may be the start of a new epidemic in Egypt [6].

Unfortunately, there are no licensed vaccines or therapeutics and no clear and definitive protocols for dealing with RVFV outbreaks. Several factors favor the repeated presence of RVFV, such as rainfall and river discharge that supports mosquitoes hatching their eggs. Climate change makes it more likely that RVFV will cross borders and reach the middle east, Europe, and the USA [7]. RVFV was isolated from more than 30 different mosquito species mainly Aedes spp. as a major vector for viral transmission, spread, and maintenance [2, 7]. Diagnosing RVFV infection is challenging as it’s a biosafety level-4 (BSL-4) with limited laboratory qualification, especially in endemic areas.

RVFV is one member of the *Phenuiviridae* family, the *Phlebovirus* genus. It is a single-stranded, enveloped virus with three segments. Large (L) and medium (M) are negative sense that encodes RNA-dependent-RNA polymerase and two glycoproteins respectively. Small (S) has an ambisense display encoding structural N protein and non-structural NSs protein. N protein is essential for the composition of ribonucleoprotein complexes (RNP) with L and has a vital role in the transcription and replication of the viral genome. Both N and L proteins are reported to be highly conserved sequences in the RVFV genome [8].

RNAi uprising the concept of gene silencing and explain the endogenous gene regulation machinery. During viral infection to plants and invertebrates, siRNA originated naturally from dsRNA intermediate and cleaved by cytoplasmic RNAse III enzyme called Dicer to produce 19–27 bp which is completely complemented to a sequence, these siRNAs then incorporated into an RNA - induced silencing complex (RISC). This incorporation then resulted in unwinding and strand separation as antisense strand still binding with RISC to complete RNA silencing by recognizing the specific target and then cutting this RNA transcript [9, 10]. siRNA silencing requires complete base pairing of small RNA resulting in target sequence cleavage by AGO2, which occurs between nucleotide 10–11 from the 5 end of the guide strand [11]. siRNA is regarded as one of the most powerful endogenous gene regulatory mechanisms, especially at post-transcription levels [12]. It can efficiently target viral transcription and inhibit replication without harming host cells [13]. This antiviral activity is mediated by a sequence-specific inhibitory pathway [14]. A significant role of siRNA is that the RNA inhibition pathway can be initialized in mammalian cells following a successful transfection of synthesized siRNA [10].

Since RNAi initial discovery in 1998 [9], it has been developed and evaluated against a wide range of viruses [15, 16]. Among the various viruses studied, several share genetic architecture,   transmission routes, and the classification of RVFV as a highly serious emerging disease such as the *Orthonairovirus* genus’s Hazara virus (HAZV), which belongs to the *Nairoviridae* family and partakes RVFV transmission vectors and genomic structure encoding N protein, two glycoproteins Gn and Gc, and L polymerase [17]. In comparison to L and M segments, siRNAs had more antiviral activity against the N mRNA expression and consequently inhibited protein synthesis [17]. Crimean-Congo haemorrhagic fever virus (CCHFV) shares the genetic structure and mode of transmission of RVFV. Nine siRNAs were developed and targeted CCHFV of the *Nairoviridae* family. In a cell-based experiment, four of the created siRNAs displayed silencing effectiveness of more than 70% when compared to the control [13]. Another case in point is when pooled siRNAs targeted the S segment of the Andes virus (ANDV), efficiently inhibiting viral infection [18]. Andes virus is classified as a Category A pathogen with serious concerns because it is the only member of the *Orthohantavirus* that can pass from person to person [19]. In a recent in vitro study against arthropod Dengue virus (DENV) infection, siRNAs demonstrated remarkable potential in silencing NS1 protein by about a 91% reduction in protein synthesis and more than 90% in viral load [20]. When siRNAs were developed against an attenuated RVFV MP-12 strain, various siRNAs targeted N protein were transfected 24 h before virus infection offered a significant protection in vitro [21]. These studies have created predictive hypotheses about the possible effects of siRNAs on RVFV infection. Last but not least, siRNA demonstrated 100% in vivo protection against the Ebola virus, which caused a terrible outbreak in 2014 in West Africa [22]. According to Bishop, this effective therapeutic achievement allowed the USA Food and Drug Administration (FDA) to grant the permission to use this siRNA for compassionate use, while it is not completely authorized for use [23]. As RVFV is endemic in Africa, the use of siRNAs against Ebola virus in West Africa may hold promise that effective siRNAs against RVFV can be used for emergency and rapid outbreaks.

These findings suggest that siRNA has the potential to be used as a novel treatment against major viral infections. The current study’s goal is to effectively construct unique siRNAs targeting diverse loci within the N mRNA transcript of RVFV and to evaluate their silencing capacities as prophylactic and antiviral treatments in vitro. The uniqueness of our investigation stems from the fact that siRNA were primarily examined when pre-transfected 24 h prior to RVFV infection and were not evaluated when siRNAs were post-transfected after RVFV infection, and the strain employed is a strain isolated from an Egyptian sheep and adapted to cell culture for use in BSL-2.

## Materials and methods

### Cell line, virus propagation, and titer determination

African Green Monkey kidney cells (Vero cells) were cultured in modified Eagle medium (MEM) from (VACSERA, Egypt) supplemented with 10% fetal bovine serum (FBS) at 37 °C with 5% CO_2_. In a BSL-2 lab, a cell-culture-adapted strain of Rift Valley fever virus (RVFV) isolated from an Egyptian sheep was propagated on 80% confluent Vero cells for 48 h. It has grown to the highest titer. The virus was collected, aliquoted, and stored at -80 °C. Titration of RVFV was proceeded on 80–90% confluent Vero cells, by performing ten-fold serial dilution on 96- well plate, virus adsorption was allowed for 1 h before the inoculum was removed, washed with PBS, and fresh media was added, cells were incubated for 48 h, CPE was monitored and TCID 50 was calculated by endpoint assay using Reed Muench equation [24] **.**

### Design, and synthesis of siRNAs

Detailed sequence analysis of the RVFV genome was done to identify the most conserved region among different strains and isolates. The RefSeq of RVFV S segment, complete genome (NC_014395.1) was downloaded from NCBI. The sequence identity of RVFV N protein was then aligned by the BLAST (http://www.ncbi.nlm.nih.gov/blast) program with 100% query coverage, using BLOUSM 62, and large word size of 28. Other programs such as LALIGN (http://www.ebi.ac.uk), and CLUSTAL OMEGA were used where the same results was obtained indicating high conservation in N protein. A 738-nucleotide sequence was used as query target for designing different siRNAs. Different online tools such as sivirus engine [25], SiDESIGN [26, 27], DISR [28], and siDirect [29], were checked for possible suggested siRNAs using first and second generation algorithms. The results were manually assessed using the Reynolds criteria [26], and guidelines proposed by El-Bashir and collaborators [30] to identify the siRNAs with the highest probability of suppressing the expression of the RVFV N mRNA. In designing and inserting our enquiry we keened to use only ORF candidates, with no SNP, avoiding internal repeats of any nucleotides more than four, and any motifs stimulating immune response. From all of these different software’s, sequences were arranged according to score, occurrence of this sequence in many different software, number of guidelines satisfied by siRNAs. BLAST tool was used to reject any sequence homology with different databases as not more than 15 consecutive nucleotides were homogenous with any unrelated gene. Top three siRNAs were developed targeting 3 different loci on N mRNA with the highest probability of blocking the expression of the RVFV (Table 1) by targeting the most conserved region of the viral genome where any mutation can lead to deviation [14]. Fortuitously, D1 siRNA was previously designed and reported [21].

**Table 1:** Sequences of siRNAs targeted Nucleoprotein

siRNA	Sequence	Position
D1siRNA	Sense: 5` gcagugaauagcaacuuuauu 3` Antisense: 5` uaaaguugcuauucacugcuu 3`	607–625
D2 siRNA	Sense: 5` gggcaauauuagaugcucauu 3` Antisense: 5` ugagcaucuaauauugcccuu 3`	488–506
D3 siRNA	Sense: 5` ggagaaggaugccaagaaauu 3` Antisense: 5` uuucuuggcauccuucuccuu 3`	150–168

### Processing of siRNAs designs

The siRNAs were provided as lyophilized 10 nmol, reconstituted in siRNA buffers according to manufacturer instructions, aliquoted in 20 µM stock solutions, and stored at -20 ° C until further use. The TOX siRNA (siTOX) (DharmaconTM RNAi technologies, Lafayette, USA) was applied in each experiment to determine the transfection efficacy and siRNA uptake into cells. A luciferase siRNA sequence from previous work [21] was utilized as the negative control. The negative control was generated and given by (DharmaconTM RNAi technologies, Lafayette, USA) to identify any changes in cells treated with various siRNAs and to compare specific and non-specific siRNA effects. Two UU overhangs were used in all the designs to boost siRNA stability against RNases [10], improving repeatability and introducing long-lasting effects even at low doses.

### Transfection efficiency using Trypan blue exclusion assay

The transfection efficacy was monitored by transfecting Vero cells with 200 nM of siTOX (Dharmacon RNAi technologies, Lafayette, USA) under the same experimental conditions. Within 24–48 h, cells that undergo efficient transfection with siTOX underwent apoptosis. These transfected cells were trypsinized and counted manually, viable cells and dead cells that retained the blue color were counted and transfection efficiency was determined. Transfection efficiency % was calculated as the percentage of viable cells in siTOX-transfected cells related to non-transfected cells. An average of 90–95% transfection efficiency was accomplished in all experiments.

### Cytotoxicity tests

A concentration-dependent MTT colorimetric assay was used in a cytotoxicity evaluation to ensure that the designed siRNAs targeted the N protein gene and did not interfere with the tested cell’s genes (off-target effect) or induce cell death. Vero cells were transfected with varying doses (varying from 2.5 nM to 100 nM) of each siRNA at 60–70% confluence. Microscopically, cells were examined 24 h after transfection. 50 µl of MTT dye was added, 50 µl of dimethyl sulfoxide (DMSO) was added to dissolve formazan crystals, and plates were examined with a microplate reader at 570 nm. The optical density (OD) of the control untreated cells, each siRNA, and the viability %, were determined. The siRNA and negative control assays were performed in triplicate. The transfection agent was also tested in a 96-well plate at a final concentration of 0.4 µl / well, as directed by the manufacturer.

A light microscopic examination was also utilized to assess siRNAs’ cytotoxic effect on Vero cells on a daily basis for 72 h. A trypan blue exclusion experiment was used to compare transfected cells to non-transfected cells.

### siRNAs antiviral activity against RVFV

Vero cells were transfected with siRNAs after 1 h of viral infection in 24-well plates to test their antiviral effectiveness. On the day of transfection, Vero cells were seeded in 24-well plates at a density of 2.5 × 10^4^ cells/well to achieve 60–70% confluent cell monolayers in a humidified incubator at 37 °C with 5% CO_2_. Cells were transfected with siRNAs at 10 nM and 30 nM doses in biological triplicates. To investigate the silencing impact on RVFV N mRNA, the following siRNAs were tested: (1) D1siRNA (2) D2 siRNA, (3) D3 siRNA, (4) pooling of D1, D2, and D3 at 30 nM as final concentration and (5) RL siRNA as a negative control.

According to the manufacturer’s instructions, the defined siRNA concentrations were complexed with the transfection reagent DharmaFECT (DharmaconTM RNAi technologies, Lafayette, USA). In brief, an equal volume of siRNA diluted with Opti-MEM reduced serum medium (30 nM and 10 nM) was mixed with a transfection reagent diluted with Opti-MEM reduced serum medium. The final volume of DharrmaFECT was 2 µL/well. The transfection mixture was incubated for 20–30 min at RT to allow the formation of siRNA-lipid complexes. The solution was then added dropwise to each well and the volumes were completed to 500 µL by media. To enhance the virus’s growth, 1% FBS was added to the medium to bring the total volume to 500 µL, as directed by the manufacturer. To optimize all assays conditions, mock-transfected cells were transfected with transfection reagent only, non-transfected Vero cells, negative control luciferase siRNA, siTOX transfection control, and cells infected with RVFV at an MOI of 2 alone were used as various controls.

For antiviral assessment, cells were infected with RVFV at an MOI of 2 for 1 h at 37 °C to allow viral adsorption with gentle shaking every 15 min to ensure uniform distribution. Following that, the viral inoculum was removed and cells were transfected with siRNA complexed with transfection reagent as stated by manufacturer instructions. After 24 h, the supernatant was collected for mRNA expression level assessment by RT-PCR and reduction in virus titer by TCID50 endpoint assay. Supernatant and pellets were collected after 48 h to determine protein expression levels using western blot. Non-transfected Vero cells that were infected with RVFV at an MOI of 2 were used as positive virus controls.

### Transfection of siRNA and evaluation of prophylactic activity

Vero cells were seeded in 24-well plates at a density of 2.5 × 10^4^ cells/well in a humidified incubator at 37 °C with 5% CO_2_ to achieve 60–70% confluent cell monolayers. In biological triplicates, cells were transfected with siRNAs at 10 nM and 30 nM doses. As previously stated, transfection mixes were prepared.

After 24 h of siRNA transfection, the incubation medium was withdrawn and replaced with new media. Cells were infected with RVRV at an MOI of 2 to measure prophylactic efficiency. Infected cells were incubated at 37 °C for 1 h to allow viral adsorption, with gentle shaking every 15 min to achieve uniform dispersion. The inoculum media was then removed and replaced with a new medium containing 1% FBS. After 24 h, inhibition of virus replication was assessed by ten-fold serial dilution of supernatant to determine log decrease in virus titer, percentage of inhibition, and the number of genome copies in cell supernatant by RT-PCR. The level of protein expression was assessed by western blot after 48 h.

### Virus titration by endpoint assay

TCID50 is one of the valuable assays for viral quantification representing virus-specific morphological changes in infected cells, leading to cell death known as Cytopathic effect (CPE). Briefly, Vero cells were seeded in 96-well plates to assess the reduction in viral titer caused by siRNA silencing activity. The supernatants from the tested siRNAs and virus controls were used. Supernatants were ten-fold serially diluted, and added to wells incubated at 37 °C and 5% CO_2_ for 48 h. During incubation, the virus is replicated and released to develop CPE. Each 96-well plate were scored for the presence or absence of CPE. TCID50 values for tested siRNAs and virus control were calculated by endpoint assay using the Reed Muench equation. Log reduction in a viral titer was determined as the difference between the endpoint dilution of each siRNAs and endpoint dilution of RVFV control. The percentage of inhibition was calculated.

### Quantitative RT-PCR detection of Rift Valley Fever Virus RNA in Vero cell line

Quantitative RT-PCR was performed to investigate the inhibitory effect of siRNA on RVFV N mRNA, and the magnitude of inhibition caused by different siRNAs against RVFV N mRNA. Viral RNA was harvested from cell culture after 24 h of viral infection; and extracted from the supernatant by Direct-zol RNA Miniprep Plus kit (ZYMO RESEARCH CORP., USA) according to manufacturer instruction protocol. The RNA elution was done in a volume of 50 µL of elution buffer and was stored at -80 ° C until further use. SuperScript IV One-Step RT-PCR kit (Thermo Fisher Scientific, Waltham, MA USA) protocol utilizing SYBR Green dye with forwarding primer **5`-GAAGGCAAAGCAACTGTGGA-3`** and the reverse primer **5`- AAGCCACTCACTCAAGACGA-3`** was used. Primers for the amplification of the N protein of RVFV were designed by Primer3 program online software to amplify 150 nucleotides of the conserved N protein. The relative amount of viral load was represented by the Ct value. Relative quantification (RQ) (2-ΔΔCT), is an important method used to calculate relative levels of gene expression and directly use the threshold cycles of target genes generated by a PCR system and normalized to the corresponding β-actin housekeeping gene. RQ is automatically generated by the PCR instrument software package.

The subsequent reaction conditions were performed as 55 °C for 10 min for reverse transcriptase activation, reverse transcriptase enzyme inactivation at 95 °C for 2 min, followed by 40 times 95 °C for 10 s, 55 °C for 15 s, 72 °C for 30 s. A final extension step at 72 °C for 5 min using the applied Biosystem (Step One Applied Biosystem, Foster City, USA).

### Determination of N protein expression level by a Western blot

To determine the effect of siRNA on the targeted N protein expression of RVFV after 48 h of transfection and infection with RVFV at an MOI = 2, western blot analysis was done as follows, Vero cells were washed with fresh PBS pH 7.4 and were re-suspended in lysis buffer which is PBS pH 7.4 containing 1% TritonX – 100 and 1X protease inhibitor. Ready PrepTM protein extraction kit was used for protein extraction. About 20 µg of total protein lysate was resolved in 2x Laemmli sample buffer containing 4% SDS, 10% 2-mercaptoehtanol, 20% glycerol, 0.004% bromophenol blue and 0.125 M Tris HCl. The pH was checked and brought to 6.8. Each mixture was boiled at 95 °C for 5 min to ensure protein denaturation before loading on polyacrylamide gel electrophoresis. The protein was transferred by electroblotting onto the PVDF membrane according to standard protocols. A blocking agent was applied for 1 h in PBS pH 7.4 containing 0.1% Tween 20 and 3% bovine serum albumin, and the blots were conjugated with mouse anti-RVFV N (R3-ID8;1:2,000) (BEIResources, NIH, Manassas, VA, USA), and anti-β-actin (1:200; Santa Cruz Biotechnology, Dallas, TX, USA) monoclonal antibodies overnight as beta-actin was used as a loading control. Then, the membranes were washed and incubated for 1 h with goat anti-mouse-HRPs conjugated secondary antibody (1:5,000) (Santa Cruz Biotechnology, Dallas, TX, USA) and specific reactivity was detected using an enhanced chemiluminescent (ECL) detection system (GE Healthcare, Buckinghamshire, UK).

### Statistical analysis

Graph pad Prism 9 was used to determine the diversity in response values between siRNAs-treated and untreated controls and to work out the statistical significance. In addition, an unpaired t-test was accustomed to compare mean response values between different siRNA treatments, and the mean values of the siRNA treatments with the controls. To be considered a statistically significant P–value ≤ 0.001. Three biological replicates were used in all experimental designs.

## Results

### Cytotoxicity of siRNA on Vero cells

**Fig. 1:**
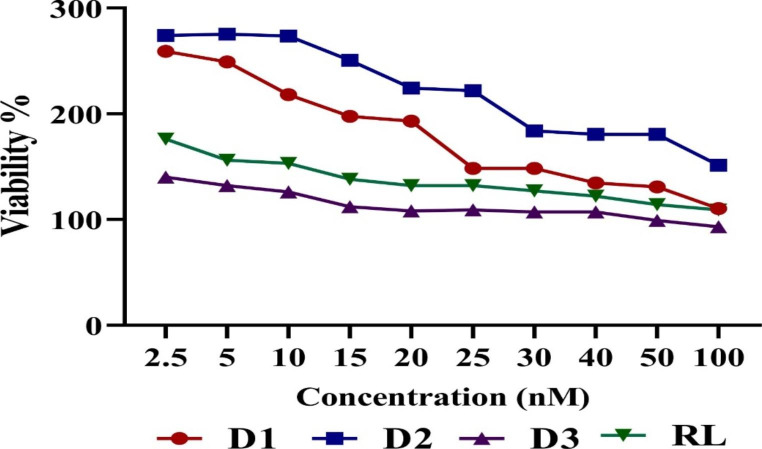
Cytotoxic effect of different siRNAs and negative control siRNA utilizing MTT colorimetric assay on Vero cell line

The non-toxic concentration of siRNAs that can be used with minimal or no toxicity on cells was evaluated by cell viability MTT assay. Microscopic visual examination of transfected cells with different siRNAs were observed after 24, 48, and 72 h of transfection. The observation of siRNAs at a concentration of 100 nM and lower shows no change in cell health or morphology. The viability % was determined in (Fig. 1). All designed siRNAs were safe, with all demonstrating high viability at 100 nM. All siRNAs that targeted RVFV N protein in S segment were used in concentrations of 30 nM and 10 nM. To confirm that the viability results were attributable to siRNA itself, the DharmaFECT transfection reagent was evaluated at the final concentration that was used in the assay (data not shown). DharmaFECT demonstrates high viability and safety to cells under the same experimental conditions.

### Microscopic examination and morphological change on Vero cells

**Fig. 2:**
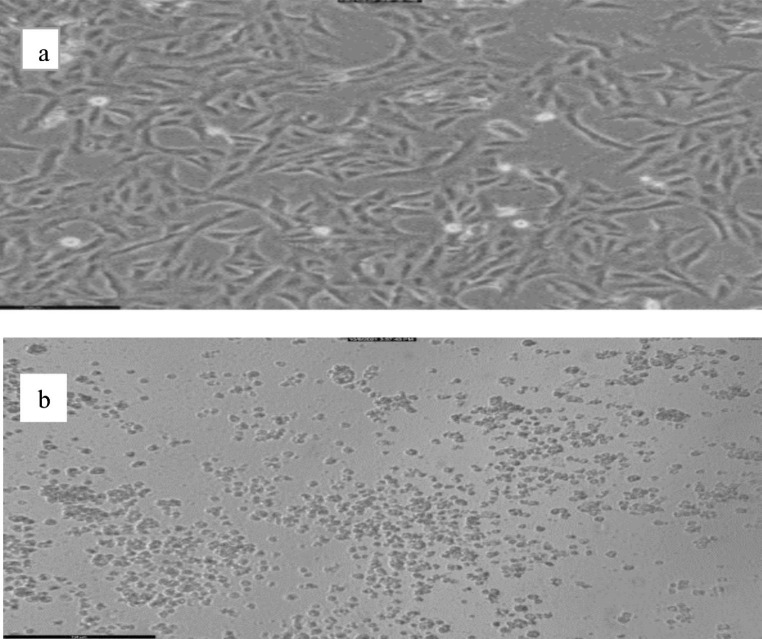
Morphological change on Vero cell line (**a**) represent Vero cells transfected with siRNAs for 72 h, (**b**) represents Vero cells infected with RVFV and cells transfected with RL negative control and RVFV

When Vero cells were monitored and examined, cells appeared healthy, homogenous, and well-adherent after 72 h, as shown in (Fig. 2a). Vero cells treated with RL negative control and infectious virus show CPE as shown in (Fig. 2b). The virus-positive control, RL siRNA negative control and non-effective siRNA show CPE, which is characterized by cell rounding, swelling, and fusion with a reduction in cell numbers per well when compared to normal Vero cells cells, while normal control cells and siRNA effective designs show no CPE with enhancing the appearance of cells and no decrease in cells number.

### Antiviral activity of different siRNAs against RVFV N protein expression on Vero cell line 

**Fig. 3:**
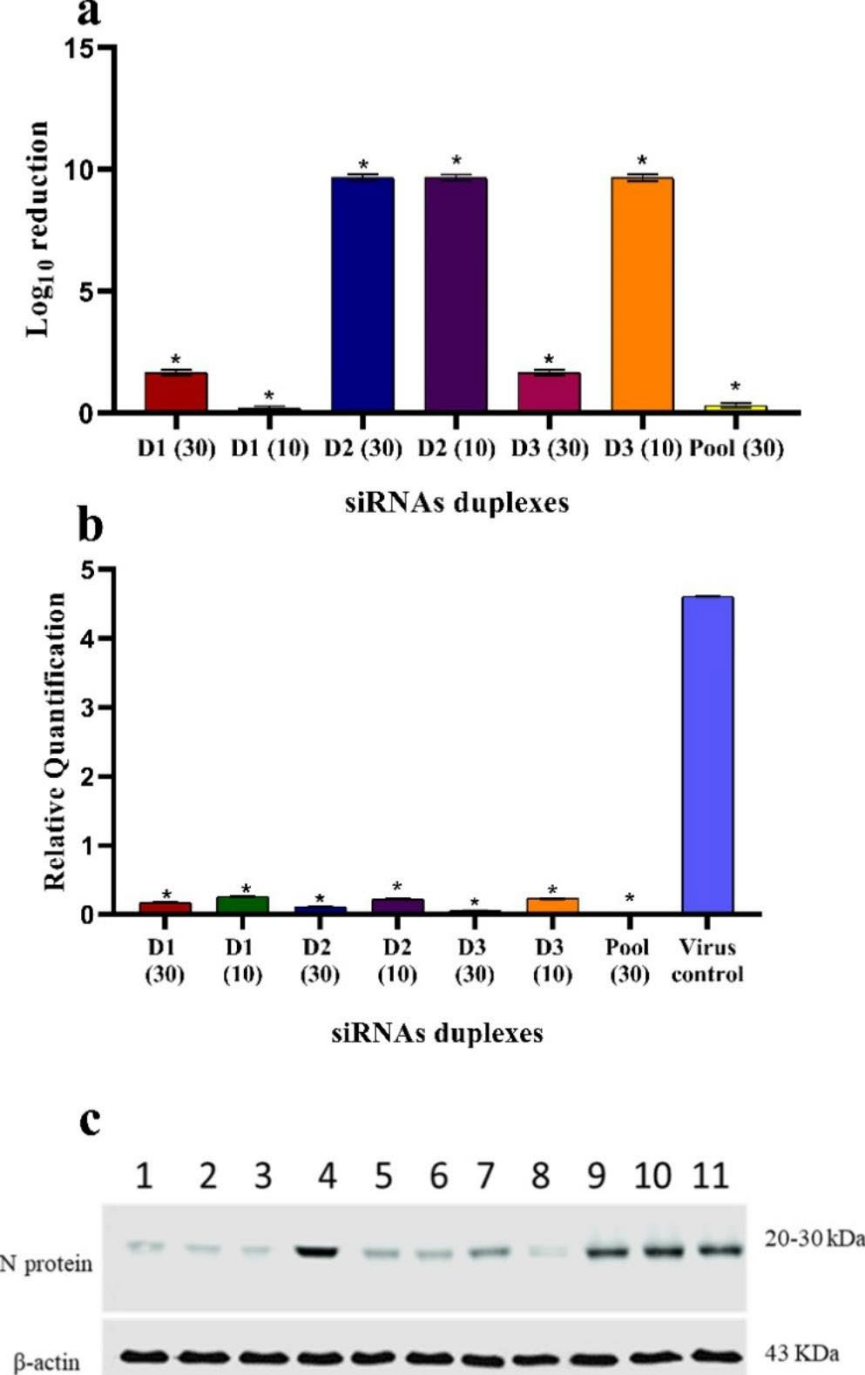
Antiviral activity of various siRNAs against RVFV cell culture adapted strain. (**a**) Illustrated log reduction (loss in virus titer) * P ˂ 0.001 indicated significance between treatments as fold reduction in RVFV titer. D2 (30 and 10 nM) and D3 (10 nM) were nearly identical and showed the highest fold reduction in virus titer. (**b**) Represents qRT-PCR inhibition analysis of different siRNAs duplexes at a concentration of 30 nM and of 10 nM against RVFV N mRNA replication. The RQ values of all siRNAs at different concentrations differed significantly from the non-treated virus control (p ˂ 0.001). As * indicate a significant difference between different treatment related to control non-treated cells. (**c**) Represent the amount of RVFV N protein when Vero cells were infected with RVFV and post-treated with different siRNAs for 48 h. Lanes 1, 2, and 3 represent D1, D2, and D3 at 30 nM concentration, lanes 4, 5, and 6 represent D1, D2, and D3 at 10 nM concentration, lane 7 represent pooled siRNAs at 30 nM concentration, lane 8 represent mock cells with transfection reagent only, lane 9, represent siRNA negative control. Lanes 10, and 11 represent virus non–treated control. β-actin was used as a loading control

All siRNAs’ designs showed inhibition in N protein expression. All siRNAs were evaluated in three biological replicates. RVFV titration was performed on the Vero cell line by TCID50 endpoint assay after 24 h of viral infection. Viral load obtained in cells infected with RVFV cell-culture adapted strain followed by treatment with specific siRNAs were significantly reduced when compared to RVFV infected control cells (*p <* 0.001) (Fig. 3a). D2 at both tested concentrations of 30 nM, 10 nM, and D3 at a concentration of 10 nM resulted in nearly complete abrogation in RVFV titer. At the same time, D1 and D3 both caused a 1.66 log reduction in RVFV titer at 30 nM concentration when compared to control untreated cells. D1 at a concentration of 10 nM and the pool of siRNAs, D1/ D2/ D3, at a concentration of 30 nM caused a 0.3 log reduction in virus titer (Fig. 3a).

After 24 h of viral infection, qRT-PCR was used to quantify the expression level of RVFV N mRNA. Supernatant from cells infected with RVFV cell-culture adapted strain under BSL-2 conditions and treated with specific siRNAs duplexes at 30 nM and 10 nM concentrations were used. Relative quantification (QR) was calculated to determine the change in N mRNA expression based on a housekeeping gene. All developed siRNAs significantly inhibited RVFV N mRNA expression when compared to non-treated control and normalized to β-actin reference control (p ˂ 0.001) (Fig. 3b). Specifically, all siRNAs’ duplexes displayed increased silencing activity when transfected after virus infection with a nearly complete abrogation of virus replication at a concentration of 30 nM by more than 96% (Fig. 3b). All siRNAs at 10 nM inhibited virus replication by more than 94%. Pooled siRNAs inhibited RVFV replication by about 99%. There was no significance between the amount of virus produced in non-treated control and RL negative controls, which confirmed the specificity and efficiency of siRNAs duplexes (data not shown).

Western blot analysis confirmed the qRT-PCR results. The level of N protein expression was inhibited when infected cells were treated with specific siRNAs at different concentrations (Fig. 3c). The silencing activity was determined by the absence of the N protein expression band (Fig. 3c). Post-treatment with D1, D2, D3 at 30 nM concentration, D2 and D3 at 10 nM concentration completely abrogate N protein expression (Fig. 3c lanes 1, 2, 3, 5, and 6). D1 at 10 nM concentration expressed N protein (Fig. 3c, lane 4).The complex pool of siRNAs inhibited RVFV N protein expression (Fig. 3c lane 7). In contrast, the N protein expression band was easily detected in the untreated controls (Fig. 3c lanes 10 and 11) as well as in RL siRNAs (Fig. 3c lane 9).

### Preventative effect of different siRNAs against RVFV N protein expression on Vero cell line 

**Fig. 4:**
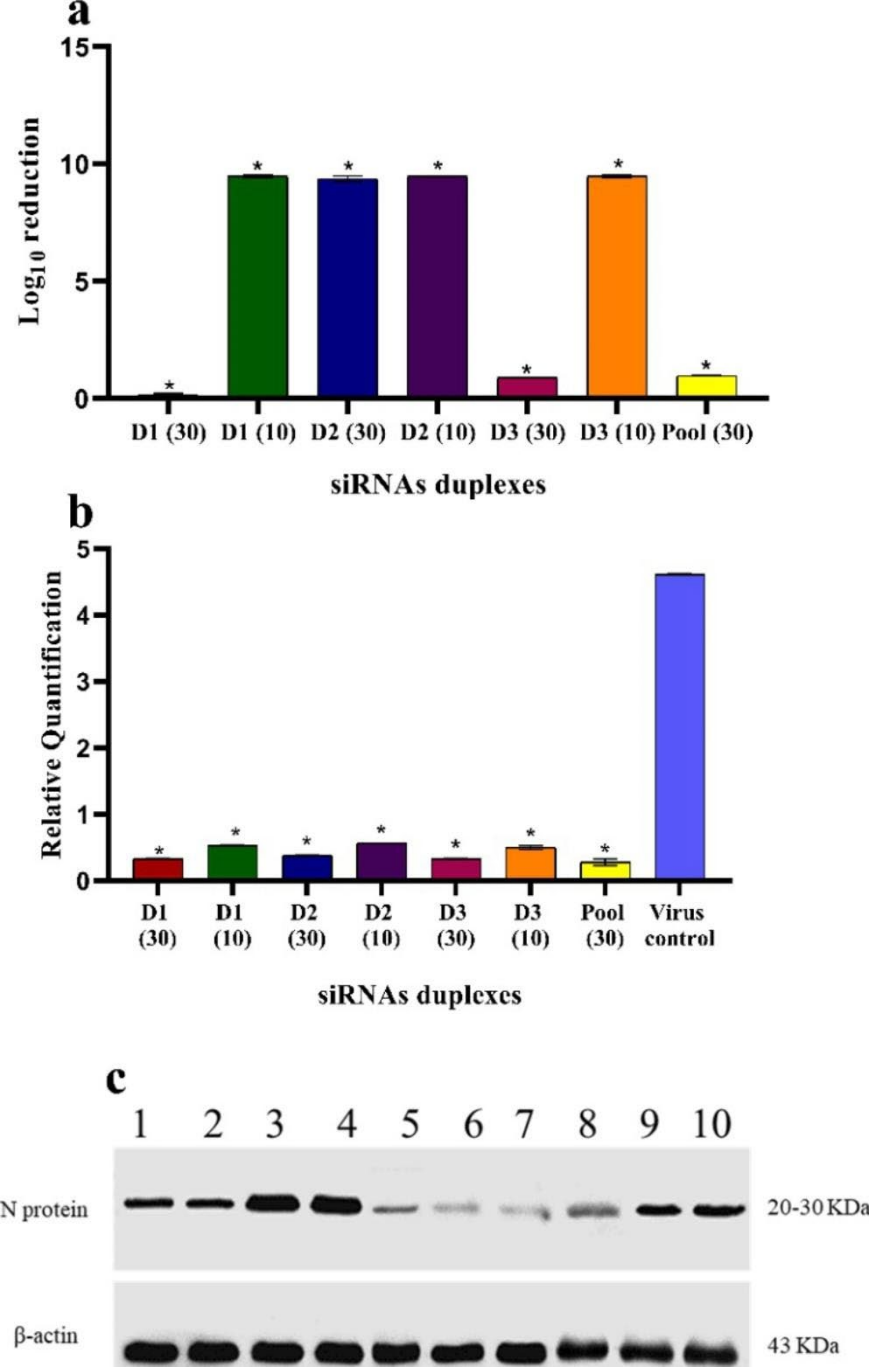
Prophylactic effect mediated by various siRNAs against RVFV cell culture adapted strain. (**a**) Represent log reduction in RVFV titer relative to non-treated virus control * P ˂ 0.001 indicated significance between treatments as fold reduction in RVFV titer. * indicates a significant difference between various treatments. (**b**) Represents q RT-PCR silencing effect of different siRNAs duplexes at a concentration of 30 nM and of 10 nM against RVFV N protein expression. The RQ values of all siRNAs at different concentrations differed significantly from the non-treated virus control (p ˂ 0.001). (**c**) Represent the amount of RVFV N protein when Vero cells were pre-transfected with different siRNAs for 72 h and infected with RVFV at MOI = 2 for 48 h. Lane 1, 2, and 3 represent D1, D2, and D3 at 10 nM concentration, lane 4, 5, and 6 represent D1, D2, and D3 at 30 nM concentration, lane 7 represent pooled siRNAs at 30 nM concentration, lane 8, 9, and 10 represent siRNA negative control and virus non-treated control, respectively. β-actin was used as a loading control

RVFV N mRNA was significantly inhibited when pre-treated with different siRNAs. All resulting data were normalized to the virus control. All siRNAs were evaluated in three biological replicates. RVFV titration was performed on the Vero cell line by TCID50 end-point assay after 24 h of viral infection. Viral load in cells pre-treated with specific siRNAs were significantly reduced when compared to RVFV infected control cells (*p <* 0.001) (Fig. 4a). D2 at both concentrations of 30 and 10 nM, D1and D3 at a concentration of 10 nM reduced RVFV titer nearly completely (Fig. 4a). Cells appeared healthy, and enhanced cell morphology by preventing virus infection and release. Whereas, D1 and D3 at a concentration of 30 nM, showed 0.2 and 0.89 log reduction in virus titer, respectively (Fig. 4a). The pool of siRNAs, D1/ D2/ D3, at a concentration of 30 nM significantly reduced viral titer by almost one log reduction. (Fig. 4a).

After 24 h of viral infection, qRT-PCR was used to investigate the prophylactic effect of siRNAs against RVFV N mRNA expression on the Vero cell lines. Relative quantification (RQ) was calculated to determine the change in N mRNA expression based on a housekeeping gene. All developed siRNAs differed significantly when compared to non-treated control and normalized to β-actin reference control (p ˂ 0.001) (Fig. 4b). Pre-transfection with different siRNAs inhibited RVFV N mRNA expression by about 92% at 30 nM and by more than 88% at 10 nM (Fig. 4b). Pooled siRNAs inhibited RVFV replication by about 95%. There was no significance between the amount of virus produced in non-treated control and RL negative controls which confirmed the specificity and efficiency of siRNAs duplexes (data not shown).

Pre-treatment with several siRNAs inhibited protein expression in Vero cells (Fig. 4c) with different levels. Pre-transfection with RL non-specific siRNA, N protein was expressed (Fig. 4c, lanes 8, and 9). The untreated virus control displayed obvious N protein expression (Fig. 4c, lane 10). siRNAs at a concentration of 10 nM (Fig. 4c, lanes 1–3), in addition to D1 at 30 nM, expressed RVFV nucleoprotein, but D2 and D3 at 30 nM, besides pooled design suppressed protein expression (Fig. 4c, lanes 5,6, and7).

## Discussion

A novel and urgent treatment solutions are necessary against emerging viral disease. RVFV is one of the most serious emerging viral infections with a significant negative influence on human and animal health. Since its first appearance in 1931, RVFV has caused successive and severe outbreaks with high mortality, especially in Africa and the Arabian Peninsula [31, 32]. RVFV epidemics cause massive economic losses in humans and livestock, as shown in Kenya during the 2007 epidemic as it set back the economy by $32 million [33]. Saudi Arabia and Yemen suffered a $90 million economic damage [34].

Several studies have shown that siRNA has a high potential as a unique and specific therapy against wide range of serious viral infections [13, 18, 20, 21, 35], whether they are positive or negative – stranded. Human RNAi-based treatment was initially created against the RSV targeted phosphoprotein [36], and siRNAs were highly effective when administered prior to viral infection. Alnylam Pharmaceutics produced ALN-RSV01, a new anti-RSV targeting nucleoprotein of the wild type of RSV. It was safe and resulted in 38% decrease in cases [37]. Lassa virus is a BSL-4 serious pathogen, siRNA has been developed and tested, indicating that siRNA can reduce virus infectivity by up to 90% [38]. Orthopoxviruses caused several human infections with varying degrees of severity ranging from mild to lethal. Monkeypox virus was found in several animal hosts in Central and West Africa exhibiting symptoms comparable to smallpox but with lesser deaths. In cell culture, two siRNA pools showed potential antiviral efficacy against MPV [39]. When the structure of the viral protein is determined, RNAi-based treatment is time and resource saving.

The purpose of this study was to assess the possibility of siRNA as a treatment approach against RVFV cell-culture adapted strain obtained from Egyptian sheep as a preventative and antiviral therapeutics to treat humans and animals before and during epidemics.

The use of RNAi as a medicine is still problematic due to many challenges in delivering siRNA to target tissue and cells, as well as inadequate cellular absorption and enzymatic degradation. The fundamental constraint is the off-target effect, however several chemical modifications aid to increase specificity and efficiency. Even so, siRNA is still a unique and encouraging therapeutic measurement due to its ease of synthesis, and adjustment that can overcome these problems [39]. Chemical modification in the sugar backbone at position 2 of the ribose ring, by addition of 2`- O-methyl or 2`-deoxy or 2`- fluor had no effect on silencing activity but enhanced plasma stability in vivo delivery, reduced non-specific effect, and escape innate immune system [40, 41]. Liposomes are one of the most common lipid-based nanoparticle carriers used as a transfection reagent for drug delivery because they are non-toxic, biocompatible, and do not stimulate the immune response [42]. SiRNA can be delivered in mammalian cell culture when conjugated with liposomes. The use of a suitable carrier has a considerable impact on the efficiency of siRNAs. In 2003, an in vivo investigation using siRNA against the nucleoprotein and polymerase acidic protein (PA) of the Influenza virus [43] resulted in a non-specific effect. In a subsequent investigation, siRNA targeting the NP and PA of the Influenza virus displayed a silencing effect when conjugated with polyethyleneimine (PEI), and these siRNAs had antiviral action when provided after viral infection [44]. One of the most encouraging in vivo results is two approved therapeutics, (patisiran) ONPATTRO® for hereditary amyloidogenic transthyretin (hATTR) amyloidosis with polyneuropathy in adults, and (givosiran) GIVLAARI™ for acute hepatic porphyria (AHP) in adults released in 2020 by Alnylam Pharmaceuticals [45]. Most recently another two approved drugs, (lumasiran) OXLUMO® for the treatment of pediatric and adult primary hyperoxaluria type 1 (PH1), and (vutrisiran) AMVUTTRA™ used for the same purpose as ONPATTRO® but different in the route of administration.

The viability assay of siRNAs against N protein in the S segment in our study ranged from 93 to 275%. The incidence of non-specific silencing activity relies on siRNA concentration, with higher concentration an off-target effect occurred [46]. Therefore, considering the MTT cytotoxicity results, the concentrations of used siRNAs were kept as low as 30 and 10 nM. When siRNAs were tested against CCHFV S, M, and L segments, viability was tested using a light microscope at various time intervals and a luminescence assay. CC50 of siRNAs against the S segment were safe at concentrations100-200 nM, CC50 of siRNAs against the M segment were safe at concentrations 100–300 nM, and CC50 of siRNAs against the L segment were safe at 100 − 55 nM. While using the lowest CC50, they utilized 10nM as the lowest concentration and 50nM as the maximum concentration [13]. The suggestion reported by the leading researchers in the siRNAs field, Dr. N. Caplen, and editors of Nature 1 was followed to optimize the assay conditions, reducing the off-target impact, and other variations in assay conditions. Using proper controls, which have the potential to improve assay results [47, 48]. siRNA targeting Renilla Luciferase sequence was used as functional non-targeting negative control and the TOX kit as a positive control in all of the experiments. Mock cells with the simply transfected agent were employed as a positive control, while cells challenged with RVFV at MOI = 2 was used as a viral control.

The antiviral impact of posttranscriptional siRNAs silencing of RVFV N protein in the Vero cell line for the first time was tested by using varying assays such as q RT-PRC, Western blot, and virus log reduction. All 3 siRNA significantly inhibited N mRNA replication. In all studies, D2 exhibited a superior antiviral silencing efficacy at concentrations of 30 and 10 nM with the absence of N mRNA expression after 48 h. The quantification of viral RNA by qRT-PCR using supernatants from different treatments revealed a significant reduction in RVFV replication (p ˂ 0.001) when compared to the untreated viral control. After post-transfection of Vero cells with different siRNA duplexes at 30 nM concentration, the maximum inhibitory efficacy was achieved, blocking virus replication. Pool siRNA effectively eliminates viral infection by around 99%. Furthermore, at a small concentration of 10 nM concentrations, all siRNA suppressed RVFV N mRNA expression significantly. After 48 h of siRNA post-treatment of Vero cells, all siRNA at different concentrations mute protein expression except for D1 at 10 nM, which may be due to insufficient transfection of D1 at 10 nM to cells. Endpoint assays revealed some variance in D1 at 10 nM and pooled siRNAs findings. These variants will be submitted to additional research.

Previously, the prophylactic effects of several siRNAs targeting RVFV MP-12 N protein were investigated and proved a superior prophylactic effect in Vitro [21]. The conventional algorithm was used to design four siRNA against N mRNA and one against L polymerase of RVFV MP-12. N mRNA expression is eliminated by a complex pool of siRNAs. Si605N and si46N have the highest silencing activity, but siRNA targeting the L polymerase gene had no effect [21]. Here, three siRNAs candidates were evaluated against RVFV cell culture adapted strain obtained from Egyptian sheep. Our findings are consistent with this work since the three tested siRNAs showed prophylactic potential against RVFV N protein. When measured using real-time PCR, D1, which is the same design as Si605N, showed considerable prophylactic activity. In our investigation, D2 had the superior inhibatory effect at 30 and 10 nM concentrations, as shown by log reduction by endpoint test. At 10 nM concentrations, D2 and D3 strongly limit RVFV replication. At 30 nM concentration, some variation was obtained in D2, D3, and pool siRNA, which requires further investigation. Even if they target the same gene, the presence of some inhibitory variance in siRNA activity is still concerning. The quantification of viral RNA by qRT-PCR using supernatants from various treatments indicated that RVFV N mRNA replication was significantly inhibited (p ˂ 0.001). Pre-transfection with 30 and 10 nM of various siRNAs duplexes resulted in high suppression of N mRNA In western blot assay, siRNAs were transfected for 72 h and showed decreased protection, particularly at 10 nM concentration as N protein was expressed. According toWu X et al. [49] these results can be explained by the fact that Vero cells doubled every 24 h, resulting in siRNAs being continually diluted over time, as well as the capacity of RVFV to spread efficiently in Vero cells, which in turn lowered silencing activity. This variation may be explained, as the dispatch of siRNA to its target cells with optimal functional concentrations varies depending on cellular processes such as siRNA endosomal concentration [50]. One possible explanation is that siRNA has a long pathway to mediate its silencing effect, with many steps before interacting with the target genome [39].

As siRNA is sufficient to decrease gene replication, RNAi represents a promising and increasing antiviral strategy, meaning that siRNA technology might be a possible therapeutic against a wide spectrum of viruses. Unlike typical antiviral therapies, siRNA can overcome antiviral resistance by targeting the most conserved area, whereas in one nucleotide alterations, redesigning a new candidate is simple. Furthermore, it will be applicable for use as a template against serious emerging viruses such as Ebola, SARS-CoV, and SARS-CoV-2 viruses. One important limitation of this study is some variance obtained in endpoint findings in D1 and D3, although this will be investigated further to determine the source of this variation.

Taken together, these findings suggest that different siRNA designs targeting the conserved N protein in the S segment of RVFV have a promising potential to eliminate RVFV infection and be a possible future therapy for one of WHO’s prioritized emerging pathogens. While the prophylactic activity of siRNA has previously been tested [21], this is the first time that the post-transfection effect of siRNAs against RVFV has been studied.

## Conclusion

This study evaluated the anti-RVFV posttranscriptional silencing activity of specific siRNAs. Different siRNAs were tested for antiviral and preventive effectiveness against RVFV, one of the most emerging viruses. All designs suppress viral replication both before and after treatment, indicating that they might be a promising antiviral innovation for RVFV during epidemics. Furthermore, the design strategy employed in this work may be utilized to create alternative siRNAs against many other viruses because it tested and achieved a significant reduction in virus reproduction, particularly with viruses that belong to the RVFV family and have the same genetic structure. In the future, the effect of these siRNAs will be investigated and assessed in vivo.

## References

[1] Dar O, Hogarth S, McIntyre S (2013). Tempering the risk: Rift Valley fever and bioterrorism. Trop Med Int Health.

[2] Pepin M (2010). Rift valley fever virus(Bunyaviridae: Phlebovirus): an update on pathogenesis, molecular epidemiology, vectors, diagnostics and prevention. Vet Res.

[3] Hassan OA (2017). The One Health approach to identify knowledge, attitudes and practices that affect community involvement in the control of Rift Valley fever outbreaks. PLoS Negl Trop Dis.

[4] Baudin M (2016). Association of Rift Valley fever virus infection with miscarriage in sudanese women: a cross-sectional study. Lancet Glob Health.

[5] Fawzy M, Helmy YA (2019) *The One Health Approach is necessary for the control of Rift Valley Fever Infections in Egypt: a Comprehensive Review*.Viruses, 11(2) 10.3390/v11020139 PMC6410127 30736362

[6] Mroz C (2017). Seroprevalence of Rift Valley fever virus in livestock during inter-epidemic period in Egypt, 2014/15. BMC Vet Res.

[7] Chevalier V (2010). Rift Valley fever–a threat for Europe?. Euro Surveill.

[8] Ikegami T (2015). Rift Valley Fever Virus MP-12 vaccine is fully attenuated by a combination of partial attenuations in the S, M, and L segments. J Virol.

[9] Fire A (1998). Potent and specific genetic interference by double-stranded RNA in Caenorhabditis elegans. Nature.

[10] Elbashir SM (2001). Duplexes of 21-nucleotide RNAs mediate RNA interference in cultured mammalian cells. Nature.

[11] Martinez J (2002). Single-stranded antisense siRNAs guide target RNA cleavage in RNAi. Cell.

[12] Corbett AH (2018). Post-transcriptional regulation of gene expression and human disease. Curr Opin Cell Biol.

[13] Földes F (2020). Small interfering RNAs are highly effective inhibitors of Crimean-Congo Hemorrhagic Fever Virus Replication in Vitro. Molecules.

[14] Dykxhoorn DM, Lieberman J (2006). Silencing viral infection. PLoS Med.

[15] Levanova A, Poranen MM (2018). RNA interference as a prospective Tool for the control of human viral infections. Front Microbiol.

[16] Levanova A (2022) *Antiviral potency of small interfering RNA molecules* In Hameed S, Rehman S, editors, Nanotechnology for Infectious Diseases., : p. p. 603–638

[17] Flusin O (2011). Inhibition of Hazara nairovirus replication by small interfering RNAs and their combination with ribavirin. Virol J.

[18] Chiang CF (2014). Small interfering RNA inhibition of Andes virus replication. PLoS ONE.

[19] Ferres M (2007). Prospective evaluation of household contacts of persons with hantavirus cardiopulmonary syndrome in chile. J Infect Dis.

[20] Villegas PM (2018). Inhibition of dengue virus infection by small interfering RNAs that target highly conserved sequences in the NS4B or NS5 coding regions. Arch Virol.

[21] Faburay B, Richt JA (2016). Short interfering RNA inhibits Rift Valley Fever Virus replication and degradation of protein kinase R in human cells. Front Microbiol.

[22] Thi EP (2015). Lipid nanoparticle siRNA treatment of Ebola-virus-makona-infected nonhuman primates. Nature.

[23] Bishop BM (2015). Potential and emerging treatment options for Ebola virus disease. Ann Pharmacother.

[24] REED LJ, MUENCH H, A SIMPLE METHOD (1938). OF ESTIMATING FIFTY PER CENT ENDPOINTS12. Am J Epidemiol.

[25] Naito Y et al (2006) *siVirus: web-based antiviral siRNA design software for highly divergent viral sequences*.Nucleic Acids Res, 34(Web Server issue): p.W448-50 10.1093/nar/gkl214 PMC1538817 16845046

[26] Reynolds A (2004). Rational siRNA design for RNA interference. Nat Biotechnol.

[27] Elbashir SM (2002). Analysis of gene function in somatic mammalian cells using small interfering RNAs. Methods.

[28] Vert JP (2006). An accurate and interpretable model for siRNA efficacy prediction. BMC Bioinformatics.

[29] Naito Y et al (2004) *siDirect: highly effective, target-specific siRNA design software for mammalian RNA interference*.Nucleic Acids Res, 32(Web Server issue): p.W124-9 10.1093/nar/gkh442 PMC441580 15215364

[30] Elbashir SM, Lendeckel W, Tuschl T (2001). RNA interference is mediated by 21- and 22-nucleotide RNAs. Genes Dev.

[31] Anyangu AS (2010). Risk factors for severe Rift Valley fever infection in Kenya, 2007. Am J Trop Med Hyg.

[32] Sow A (2014). Rift Valley fever outbreak, southern Mauritania, 2012. Emerg Infect Dis.

[33] Rich KM, Wanyoike F (2010). An assessment of the regional and national socio-economic impacts of the 2007 Rift Valley fever outbreak in Kenya. Am J Trop Med Hyg.

[34] Al-Afaleq AI, Hussein MF (2011). The status of Rift Valley fever in animals in Saudi Arabia: a mini review. Vector Borne Zoonotic Dis.

[35] Maffioli C (2012). SiRNA inhibits replication of Langat virus, a member of the tick-borne encephalitis virus complex in organotypic rat brain slices. PLoS ONE.

[36] Bitko V (2005). Inhibition of respiratory viruses by nasally administered siRNA. Nat Med.

[37] Alvarez R (2009). RNA interference-mediated silencing of the respiratory syncytial virus nucleocapsid defines a potent antiviral strategy. Antimicrob Agents Chemother.

[38] Müller S, Günther S (2007). Broad-spectrum antiviral activity of small interfering RNA targeting the conserved RNA termini of Lassa virus. Antimicrob Agents Chemother.

[39] Alkhalil A (2009). Inhibition of monkeypox virus replication by RNA interference. Virol J.

[40] Juliano RL (2016). The delivery of therapeutic oligonucleotides. Nucleic Acids Res.

[41] Anjorin M (2019) *The use of antimicrobial peptides, LL-37 and derivatives, to target Rift Valley Fever Virus infection*.Master of Science Biology. George Mason University, https://hdl.handle.net/1920/11601

[42] Narenji M, Talaee MR, Moghimi HR (2016). Investigating the effects of size, charge, viscosity and bilayer flexibility on liposomal delivery under convective flow. Int J Pharm.

[43] Ge Q (2003). RNA interference of influenza virus production by directly targeting mRNA for degradation and indirectly inhibiting all viral RNA transcription. Proc Natl Acad Sci U S A.

[44] Ge Q (2004). Inhibition of influenza virus production in virus-infected mice by RNA interference. Proc Natl Acad Sci U S A.

[45] Hu B (2020). Therapeutic siRNA: state of the art.

[46] Caffrey DR (2011). siRNA off-target effects can be reduced at concentrations that match their individual potency. PLoS ONE.

[47] Whither (2003) *RNAi?* Nat Cell Biol, 5(6): p. 489 – 90 10.1038/ncb0603-490 12776118

[48] Huppi K, Martin SE, Caplen NJ (2005). Defining and assaying RNAi in mammalian cells. Mol Cell.

[49] Wu X (2010). Inhibitory effect of small interfering RNA on dengue virus replication in mosquito cells. Virol J.

[50] Oliveira S, Storm G, Schiffelers RM (2006). Targeted delivery of siRNA. J Biomed Biotechnol.

